# A Narrative Review of Periodontal Vaccines: Hope or Hype?

**DOI:** 10.7759/cureus.80636

**Published:** 2025-03-15

**Authors:** Pratiksha Patel, Bhavin Patel, Shruti D Vyas, Maitri S Patel, Tanvi Hirani, Mainul Haque, Santosh Kumar

**Affiliations:** 1 Department of Periodontology and Implantology, Karnavati School of Dentistry, Karnavati University, Gandhinagar, IND; 2 Department of Pharmacology and Therapeutics, National Defence University of Malaysia, Kuala Lumpur, MYS; 3 Department of Research, Karnavati School of Dentistry, Karnavati University, Gandhinagar, IND

**Keywords:** adaptive immunity, antigenic targets, bacterial virulence factors, gingival inflammation, host modulation therapy, immunization strategies, oral microbiome, periodontal pathogenesis, prophylactic approaches, therapeutic interventions

## Abstract

Globally, periodontal diseases, mainly driven by polymicrobial biofilms, are a widespread concern of social medicine due to their considerable incidence and tie-up to systemic disorders like diabetes, cardiovascular diseases, and complications during pregnancy. Traditional treatments focus on mechanical debridement and antimicrobial therapies, but these approaches have limitations, including recurrence and antibiotic resistance. Periodontal vaccines offer a promising alternative by targeting the immunological mechanisms underlying periodontal disease. This review explores the current state of periodontal vaccine development, highlighting key antigens, vaccine delivery systems, and preclinical and clinical advancements. Special emphasis is placed on antigen selection, host variability, immune tolerance, and future directions to overcome these barriers. This article highlights the advancements and challenges in periodontal vaccine research, offering insights into the capability of immunoprophylaxis as a groundbreaking way to manage periodontal diseases.

## Introduction and background

The realm of exploration of the human microbiome is to elucidate the impact of microorganisms on the physiological functions of their host [1]. The microbiome and host shape the central constituents of the innate and adaptive immune arrangements [2,3]. Diseases and disruption of body systems can result from microbial dysbiosis [1]. The rima oris, the place of residence to over 700 bacterial species, contains the secondmajor and most multifarious microbiota after the gut, accommodating a range of microorganisms such as protozoa, fungi, bacteria, and viruses [4]. These include several pathogenic organisms, such as *Streptococcus mutans*, *Porphyromonas gingivalis*, *Tannerella forsythia*, and *Aggregatibacter actinomycetemcomitans*, which contribute key roles in the development of dental caries and periodontal disease [1].

Oral biofilms cause periodontal diseases [5]. Furthermore, not all microorganisms within the biofilm are pathogenic; certain microbes are helpful to us. These beneficial microbial communities are imperative in maintaining an interaction between pathogens and beneficial microbes. Hence, the host's immune-inflammatory system was triggered or stimulated, inhibiting plaque microbiome formation and preventing dysbiosis [6]. According to the long-standing paradigm, the oral microbiota changes from being composed mainly of gram-positive aerobes to primarily of gram-negative anaerobes, such as periodontitis [7]. One novel research study has shown that disturbances in the oral microbiome can lead to periodontitis onset [8].

Oral pathogens may also migrate into the bloodstream, affecting systemic health and chronic multi-system disorders, e.g., cardiovascular disease and diabetes mellitus [9,10]. Currently, periodontal disorder is now recognized as the sixth complication of diabetes, influencing its onset, progression, and management [11]. Periodontal pockets develop due to the disintegration of collagen strings in the periodontal nexus of ligature. Consequently, adverse systemic ramifications may arise due to deleterious endotoxins and exotoxins infiltrating the circulatory system via the compromised epithelium of the periodontal pocket [12]. This bacteremia stimulates systemic immune responses and exacerbates inflammation, impairing metabolic control and insulin sensitivity [13]. Persistent periodontal inflammation can dysregulate glucose metabolism by impairing the effectiveness of insulin, thereby exacerbating hyperglycemia [14].

Periodontal pathogenic microbes like *Porphyromonas gingivalis* and *Aggregatibacter actinomycetemcomitans *can enter the bloodstream, where they, along with their endotoxins (such as lipopolysaccharides), may directly infect vascular tissues or trigger a systemic immune response, accelerating the development of atherogenesis [15]. Preventive strategies are widely recognized as alleviating the prevailing and notable diseases impacting the mouth, including gingivitis, periodontitis, dental caries, and oral cancer [2].

At present, a variety of methodologies are available for managing periodontal disease [3]. These involve the physical elimination of biofilm by utilizing nonsurgical or surgical periodontal treatments [16] and adjunctive strategies such as administering antimicrobials below the gum line [4] or employing sub-antimicrobial dose doxycycline to modulate host response therapy [5]. Contemporary treatment modalities have thus far been unable to achieve a complete resolution of the disease or avert its recurrence; they have merely succeeded in impeding its progression [6].

Problem statement of this study

Contemporary therapeutic approaches predominantly concentrate on alleviating symptoms instead of targeting the fundamental etiology of periodontal inflammation. A periodontal vaccine that modulates the host immune response to periodontal pathogens presents a promising alternative [17]. This review explores the potential of periodontal vaccines in preventing and managing periodontal disease, evaluating their mechanisms, clinical efficacy, challenges, and prospects. It also examines the latest research and innovations in vaccine development for periodontal health.

Objectives of the study

The authors intended for this narrative review to critically evaluate the capability of periodontal vaccines as a preventive and medicinal strategy for periodontal diseases, focusing on their ability to modulate the immune response against the periodontal pathogen. It will also review the current scientific evidence on periodontal vaccines' mechanisms, efficacy, and safety, highlighting preclinical and clinical trial data. We also plan to identify the challenges and prospects in developing periodontal vaccines, providing insights into emerging research and innovations in periodontal health.

## Review

Materials and methods

**Figure 1:**
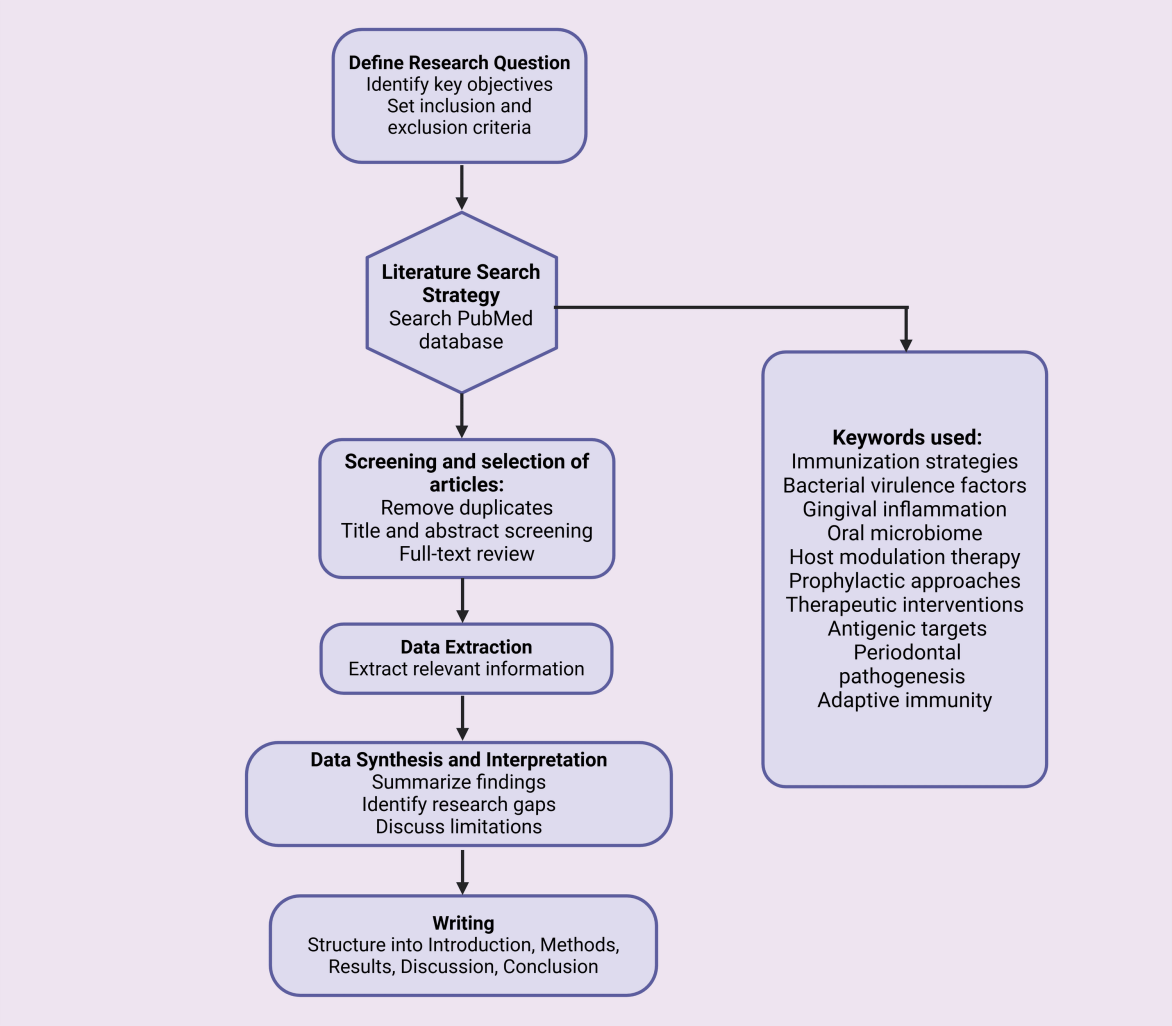
A flow chart illustrating the methodology of the paper This figure was drawn using the premium version of BioRender [18] (https://BioRender.com/l44w283)  accessed on March 1^st^, 2025, with license number OX27Z00QI0. Image credit: Pratiksha Patel

We conducted this review by searching relevant literature from PubMed, Scopus, and Google Scholar databases. Keywords were "Immunization strategies," AND "Gingival inflammation," AND "Oral microbiome," AND "Bacterial virulence factors," AND "Host modulation therapy," AND "Antigenic targets," AND "Adaptive immunity," AND "Prophylactic approaches," AND "Therapeutic interventions," AND "Periodontal pathogenesis" were used to identify peer-reviewed articles published. We critically analyzed and synthesized the articles to provide a comprehensive overview. Data were organized thematically to highlight key trends, challenges, and future directions. Figure 1 portrays the flowchart illustrating the methodology of this paper.

Review of literature

How Does Immunity Develop?

The immunological response is a consequence of the process in which an antigen, either directly or through antigen-presenting cells, identifies lymphocytes and differentiates into effector cells and memory cells specific to that antigen [18]. This response manifests in two forms, namely humoral and cell-mediated [19,20]. The generation of antibodies by plasma cells in the bloodstream is crucial for humoral immunity [21]. In contrast, cell-mediated immunity mainly depends on developing T cells (a category of white blood cells termed lymphocytes) that show specific responsiveness to the stimulating agent and are generally effective against intracellular pathogens [22,23]. The antibodies generated by effector B lymphocytes upon initial exposure to an antigen are typically short-lived [24,25]. They exhibit a gradual increase and rapid decline of immunoglobulin levels in the serum, termed the primary response. Subsequently, memory cells possibly activated. Subsequently, memory cells can be activated. During subsequent encounters with the same antigen, leading to a rapid and sustained production of antibodies, known as the secondary response [26,27]. This response can occur through various mechanisms, including neutralizing microbes and toxins, facilitating opsonization and phagocytosis of microbes, promoting antibody-dependent cellular cytotoxicity, or enabling the phagocytosis of microbes tagged with complement fragments like complement component 3b (C3b) is a polypeptide working for the inherent immune set-up [28-30].

Pathogenesis of Periodontitis

Periodontitis has a complex cause encompassing interactions among the host, microorganisms, and environmental factors, including genetic components [31]. Periodontal tissues harbor over 300 microorganisms, with certain species identified as the primary pathogens responsible for periodontitis: *Porphyromonas gingivalis*, *Aggregatibacter actinomycetemcomitans*, and *Tannerella forsythia* [32,33]. These bacteria generate various antigens that activate pro-inflammatory cells, triggering the release of multiple cytokines. These antigens activate T helper type 1 (Th1) or T helper type 2 (Th2) cells [34,35]. Dendritic cells internalize antigens and present them to cluster of differentiation 8 (CD8) or CD4 T lymphocytes associated with major histocompatibility complex (MHC) molecules [7,36,37]. Activated CD8 cells add to a Th1 response by liberating cytokines like interferon-gamma (IFN-γ), which support the progress of Th1 helper cells, resulting in a robust cell-mediated immunity (CMI) response in opposing intracellular pathogens [38-40]. CD4 cells trigger a Th2 response, producing an antibody-mediated response that provides protection [41-44].

**Figure 2:**
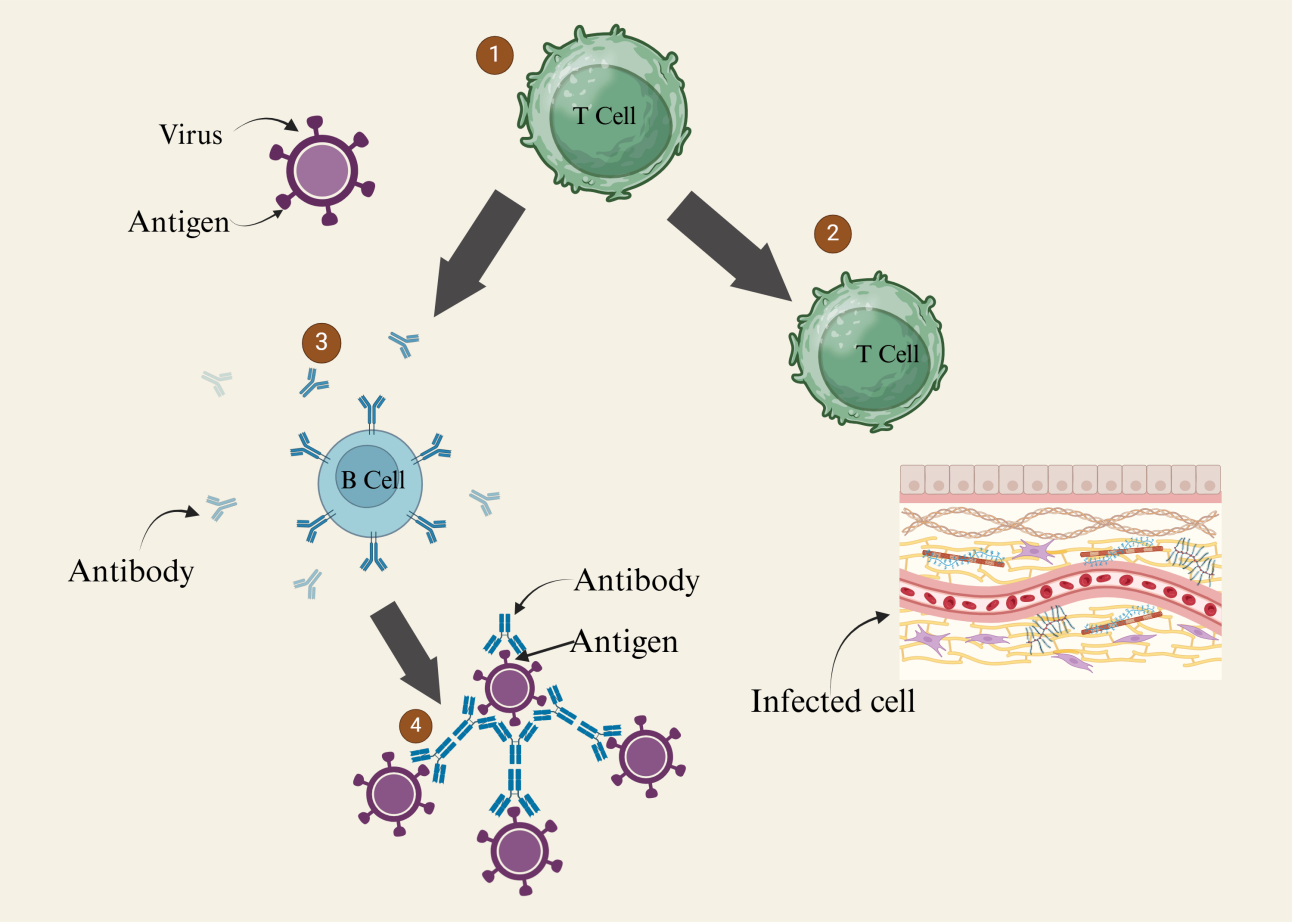
Immune response T Cell: T-cells are a type of white blood cell called lymphocytes; B Cell: B lymphocytes (bursa-derived cells). This figure was drawn using the premium version of BioRender [18] (https://BioRender.com/)  accessed on January 21^st^, 2025, with license number IU27TH5GP8. Image credit: Pratiksha Patel

Defensins, cathelicidins, and saposins are examples of antimicrobial peptides produced by the host. They play a vital role as the initial protective mechanism in opposing microbial elements while preserving the integrity of host tissues (Figure 2) [45, 46]. Nevertheless, bacterial virulence factors can counter these antimicrobial peptides' efficacy [47]. Upon breaching this initial defense, a cascade of cytokines is generated, with the potential to be either pro-inflammatory or anti-inflammatory. Inappropriate cytokine production is implicated in the development of periodontitis [8, 48, 49].

Need for Development of the Periodontal Vaccine

The oral cavity and nasal passages serve as the primary points of ingress for infectious pathogens and allergens into the human organism [50,51]. Around 66% of all pathogens gain access to the human body through these specific routes [52]. The requirement for the development of vaccines has arisen because of the subsequent factors [53,54].

Bacterial Species That Possess the Ability to Circumvent Host Immunological Defenses and Infiltrate Various Tissues

*Porphyromonas gingivalis* produces proteases that serve a dual purpose [55,56]: They supply peptides essential for growth and disintegrate serum antibacterial constituents and immune cell-originated peptides, thus hindering the activation of an antibacterial immune response [10,57,58]. Furthermore, these bacteria can avoid parts of the immune system in the gums by penetrating epithelial cells. They can also get into the bloodstream by invading endothelial cells [11,55,59].

Another virulent pathogenic bacterium causing periodontitis, *Aggregatibacter actinomycetemcomitans* [60], has unique traits, including the ability to yield a protein (leukotoxin) that targets affected individuals' immune cells like neutrophils and monocytes, as well as the capacity to produce substances that suppress immune responses, thereby ensuring their survival [12,60,61]. Furthermore, *Aggregatibacter actinomycetemcomitans* can infiltrate both epithelial and endothelial cells. These cellular structures are instrumental in establishing a favorable milieu that protects the pathogen, facilitates its ingress into the circulatory system, and acts as a springboard for transmission to additional organs [13,60,62]. Therefore, periodontal pathogens' ability to evade immune system components allows them to multiply and accumulate to critical levels, creating an environment that promotes the initiation of harmful disease processes following infection [14,31,63].

To Mitigate the Prevalence of Systemic Diseases That Are Concomitant With Severe Gum Diseases

Severe gum and bone diseases around the teeth have been extensively documented as conditions that affect the tooth and its supporting tissues and lead to a range of systemic consequences [9,64,65]. A raised quantity of inflammatory features, e.g., C-reactive protein (CRP) [66] and fibrinogen [67], have been noted in individuals with periodontal disorders. These systemic transformations increase the risk of various non-communicable disorders (NCDs) and communicable diseases that include myocardial infarction, cerebrovascular stroke, premature low birth weight babies, and pneumonia [68]. There may also be a connection at the microbial level. Research shows that the heat shock protein (HSP) antigen of *Porphyromonas gingivalis* functions as a major antigen in other microorganisms [15].

The relationship between HSP-60 ((GroEL) is an acronym for the protein GroEL) and atherosclerosis, as well as *Chlamydia pneumoniae* infection, has been established [69]. Multiple empirical studies indicate that individuals with higher levels of anti-HSP antibodies, e.g., HSP-60, DNA-K (a protein part of a cellular chaperone machinery), and GroEL, are more likely to exhibit better periodontal tissue health [30, 70-72]. As a result, the possibility of creating a vaccine for periodontitis using *Porphyromonas gingivalis*, specific HSP, or HSP epitopes appears promising.

Financial Aspects of Periodontal Diseases

Periodontal disease management entails a significant economic burden on affected individuals [73-75]. A vaccine to prevent or alleviate periodontal disease would offer considerable advantages in developing and developed nations [53,76].

Mechanism of Action of the Vaccine

**Figure 3:**
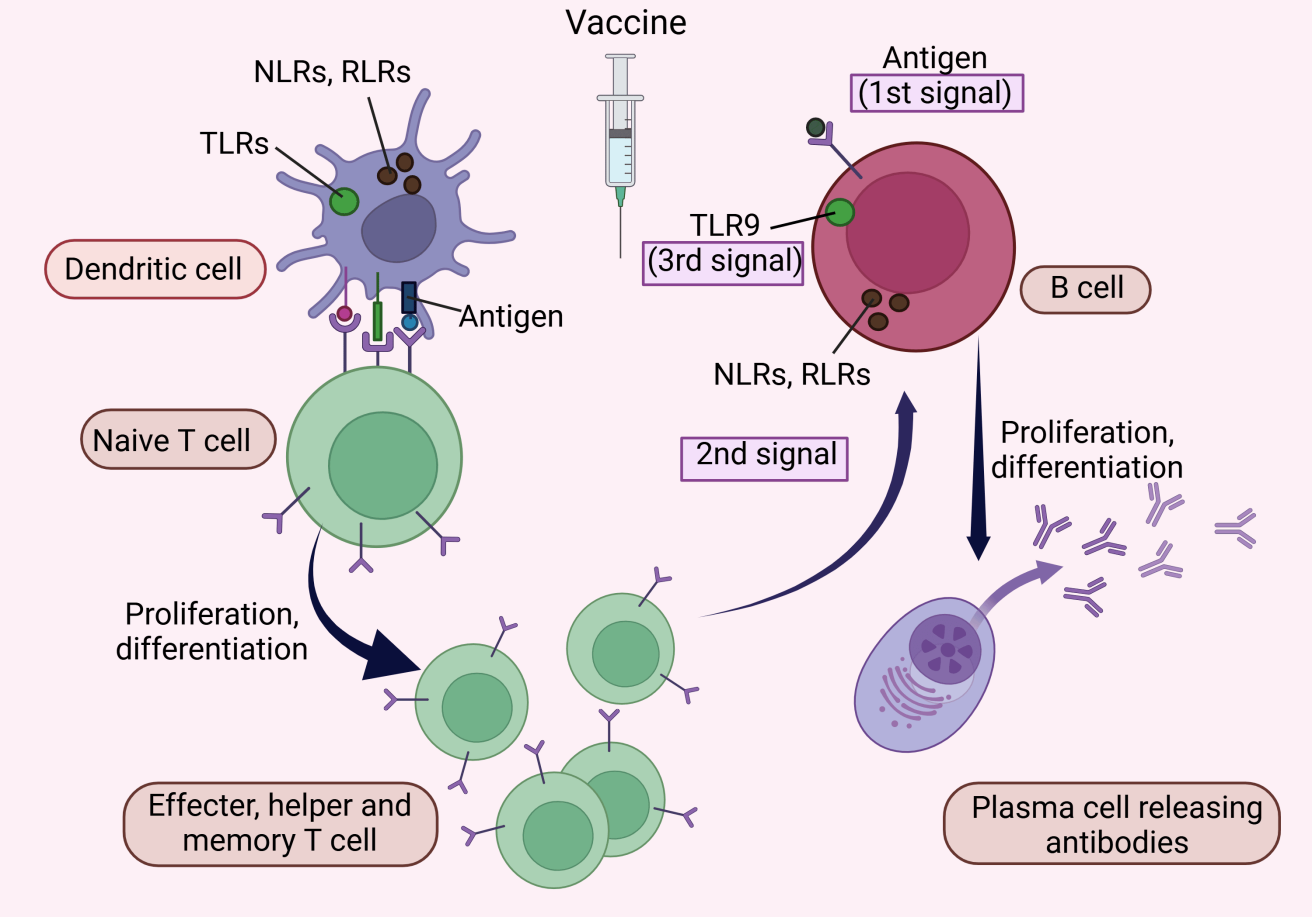
Development of an effective vaccine NLRs: nucleotide-binding oligomerization domain-like receptors; RLRs: retinoic acid-inducible gene-I-like receptors; TLRs: toll-like receptors; Naive T cell refers to a type of T cell that has fully matured in the thymus but has not yet encountered its specific antigen; Memory T cell stands for a kind of white blood cell called a T lymphocyte; B cells are B lymphocytes (bursa-derived cells). This figure was drawn using the premium version of BioRender [18] (https://BioRender.com/) accessed on 21st January, with license number QW27TH2NKD. Image credit: Pratiksha Patel

At the most basic level, a vaccine necessitates the presence of an antigen coupled with an adjuvant (with live, attenuated vaccines inherently containing the adjuvant) [77-79]. The generation of antibodies in response to the vaccine depends on two specific signals required by B cells: (i) The attachment of the antigen to the cell membrane-bound receptor, along with (ii) a co-stimulatory signal delivered by T cells activated by dendritic cells presenting the same antigen, are essential processes [37,80]. Adjuvants play a vital role in providing this second signal. Specific adjuvants, like toll-like receptor 9 (TLR9) agonists, can offer an additional stimulatory signal by targeting TLR9 in B cells (lymphocytes) [81]. Moreover, some nucleotide-binding oligomerization domain (NOD)-like receptors (NLRs) and retinoic acid-inducible gene I (RIG-I)-like receptors (RLRs), which are believed to be expressed in various immune cells, can induce cell activation, making them promising candidates for developing new vaccine adjuvants [82,83]. This mechanism is depicted in Figure 3.

**Figure 4:**
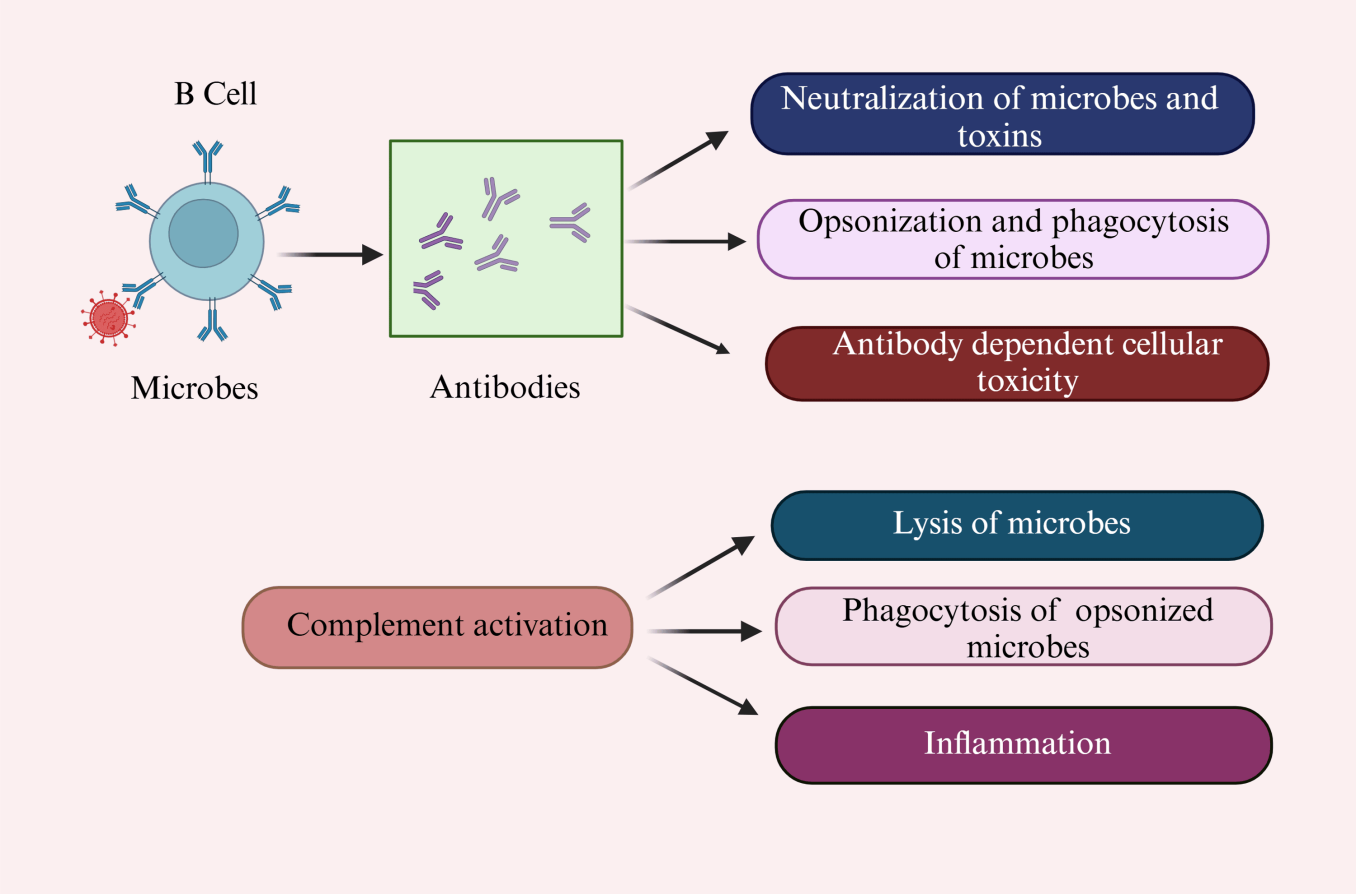
The vaccine's mechanism of action B cells: B lymphocytes (bursa-derived cells) This figure was drawn using the premium version of BioRender [18] (https://BioRender.com/) accessed on 21^st^ January, with license number PF27TH0NW2. Image credit: Pratiksha Patel

Antibodies have different functions. They neutralize foreign substances such as pathogens and toxins by binding to them and secreting them into the blood and mucosa [84]. They also activate the complement system to destroy bacterial cells by lysis (punching holes in the cell wall) [85]. They also facilitate the phagocytosis of foreign substances by phagocytic cells (opsonization) [86,87]. Figure 4 depicts the vaccine's mechanism of action.

Types of Immunization

Immunization is classified into active, passive, and genetic [88]. Active vaccination relies on introducing whole bacterial cells, subunit vaccines, or synthetic peptide antigens to activate the immune system [89,90]. In contrast, passive immunization delivers ready-made antibodies, such as murine monoclonal antibodies or plantibodies, to offer rapid protection [91]. Genetic immunization, a more advanced method, uses technologies like plasmid vaccines or live viral vectors to trigger an immune response [92,93].

Active Immunization for Periodontal Disease

This process stimulates an individual's immune system by introducing killed or live attenuated materials derived from microorganisms [94]. An overview of the results from animal research models used in active vaccination trials for periodontitis [95]. Active immunization was conducted using intact microbial cells, outer constituents, or laboratory-made polypeptides [96], or as immunogens [97].

Porphyromonas gingivalis

Whole bacterial cell: In one study, entire *Porphyromonas gingivalis* cells were used. Rats immunized with *Porphyromonas gingivalis* cells and partially purified fimbriae from the same bacterium showed elevated serum antibody levels [6,66]. The proteinase's cysteine and collagenase activity was also reduced in periodontal tissue damage [98,99]. Researchers have conducted active immunization using intact microbial cells, outer constituents, or laboratory-made polypeptides as immunogens [100].

Subunit vaccines (SV): These can be made using virulence factors such as gingipains implicated in periodontal pathogenesis [101]. Researchers have utilized the virulence factors of *Porphyromonas gingivalis* as key elements in developing active immunization (Table 1) [102]: i. outer membrane protein, ii. capsular lipopolysaccharide, iii. gingipains, iv. fimbriae, and v. HSP.

**Table 1:** Summary of findings from animal study models for active immunization vaccine trials in periodontitis Table credit: Pratiksha Patel

Bacteria	Virulence factor	Author
Porphyromonas gingivalis	Whole-cell	Persson et al., 1994a, 1994b [103,104]
	Fimbriae	Evans et al., 1992 [94], Han et al., 2014 [105]
	Gingipains	Moritz et al., 1998 [106]
	Gingipains R	Genco et al., 2004 [107]
	Gene vaccine	Ross et al., 2004 [108]
	Specific Immunoglobulin	Gibson 3^rd^ et al., 2001 [109]
	RgpA-Kgp proteinase-adhesin complexes	Rajapakse et al., 2002 [110]
	Serum antibodies	DeCarlo et al., 2003 [111]
Actinobacillus actinomycetemcomitans	Leukotoxin	Ebersole et al., 1990 [112]
*Fusobacterium nucleatum* and *Porphyromonas gingivalis* in combination	Th1/Th2 cytokine	Gemmell and Seymour, 2004 [113]

Outer membrane protein (OMP): The transcutaneous administration of a 40kDa (denotes a protein or molecular structure that has a molecular weight of 40,000 Daltons) outer membrane protein has proven effective in preventing the co-aggregation of *Porphyromonas gingivalis* with *Streptococcus gordonii* [114,115]. Moreover, this method shows potential for advancing vaccine development targeted at passive immunization. The polyclonal anti-40kDa OMP antibody has shown potential for generating a protective response through complement-mediated bactericidal activity [116,117].

Capsular lipopolysaccharide: A crucial element in assessing the pathogenicity of *Porphyromonas gingivalis*, a periodontal pathogen, is the capsular polysaccharide (CPS) it generates. Gonzalez and colleagues conducted a study [118]. This study group separated the *Porphyromonas gingivalis* CPS, gave it to mice for immunization, and utilized a murine oral challenge model to assess the *Porphyromonas gingivalis* CPS's effectiveness as a possible vaccination. In animals immunized with *Porphyromonas gingivalis* CPS, there was an upsurge in the quantity of immunoglobulin M (IgM) and IgG in the blood, and these antibodies exhibited reactivity to intact *Porphyromonas gingivalis* cells. Oral bone deterioration caused by *Porphyromonas gingivalis* was prevented in the rodents who received the CPS vaccination. These results highlight *Porphyromonas gingivalis* CPS as a viable option for a vaccination intended to stop *Porphyromonas gingivalis*-induced oral bone loss [118].

Melssen et al. [119] discovered that encapsulated *Porphyromonas gingivalis*caused more severe infections in a murine model. Another study [120], supported this finding, showing that experimental animals exposed to capsulized *Porphyromonas gingivalis *developed more severe infectious issues than those exposed to non-capsulized strains.

Fimbriae: ﻿Chaplin in 2010 reported that the components found on the surface of cells serve as crucial antigens, representing the most sophisticated immunogens [121]. The functions of fimbriae can be outlined as follows: Firstly, they facilitate adherence to the host, which marks the initial stage in the pathogenicity of microorganisms [122]. Fimbriae can attach to hydroxyapatite coated with saliva, thereby fastening *Porphyromonas gingivalis* to the outer membrane [123]. In addition, fimbriae play a role in the incursion of oral epithelial cells and fibroblasts, and they also affect inflammation by stimulating the release of interleukin (IL-1α) and tumor necrosis factor (TNF) [10].

Xu et al. (2020) revealed that fimbriae possessing a cementing function from *Porphyromonas gingivalis* are outer membrane structures that could play a considerable role in the pathogenicity of this oral pathogen, making them a promising target antigen [56]. In an experimental study using *Porphyromonas gingivalis*-infected gnotobiotic rats, vaccination with an exceedingly refined 43-kDa (kilodaltons) fimbrial protein showed a protective effect against damage to periodontal tissues [94]. Conversely, immunization with a comparably purified 75-kDa surface molecule did not yield the same level of protection [94]. Importantly, heat-treated intact cells and disrupted surface extracts containing both the 43-kDa protein and the 75-kDa component also showed defending effects. This study highlights the potential of the fimbrial protein as a promising candidate for developing vaccines to fight periodontitis, a widespread oral disease in humans [94].

Gingipains: Plaza et al. (2016) revealed that these enzymes are cysteine proteinases with trypsin-like characteristics secreted by *Porphyromonas gingivalis*, a major contributor to adult periodontitis [99]. They break down man-made and biological substrates at arginine or lysine residues and are denoted as arginine gingipain (Rgp) and lysine gingipain (Kgp), respectively [124]. Into et al. (2006) reported that arginine-specific gingipains belong to groups A and B [125].

Pathogenic Activities of Gingipains

**Figure 5:**
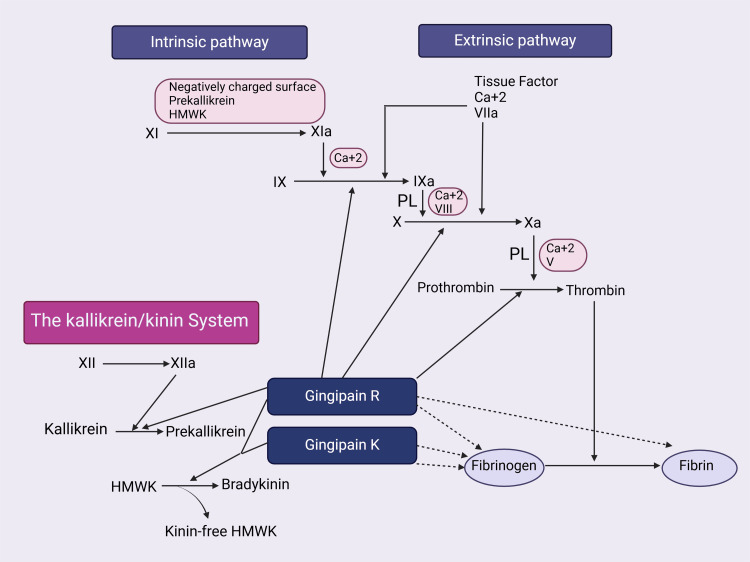
The target sites of gingipains in the kallikrein/kinin and clotting system. Ca^2+^: Calcium; XI: Plasma thromboplastin antecedent (PTA); XIa denotes a blood coagulation protease that contributes to thrombin generation; IX: Christmas factor, is one of the serine proteases involved in coagulation; IXa: A protein that helps blood clot by activating factor X; X: A protein in the blood that helps blood clot. It's also known as Stuart-Prower factor; Xa: An activated enzyme that plays a key role in blood coagulation. It's also known as thrombokinase; XII: Known as Hageman factor, is a protein in the blood that helps it clot; XIIa: An enzyme that helps blood clot, and is also involved in fibrinolysis and the production of bradykinin and angiotensin; PL: platelet phospholipid This figure was drawn using the premium version of BioRender [18] (https://BioRender.com/) accessed on 21^st ^January, with license number TV27TH3MKH. Image credit: Pratiksha Patel

Instigation of the kallikrein/kinin system: Schmaier and McCrae (2007) reported that plasma kallikrein is formed from prekallikrein by the action of activated Hageman factor, which results in the release of bradykinin from high-molecular-weight kininogen [126]. Imamura et al. (2003) reported that bradykinin enhances vascular permeability [99]. Arginine-specific gingipain (HRgpA) and HRgpB (referred to as a hydroxyproline-rich glycoprotein (HRGP)) that produce bioactive C5a (denoting a bioactive particle that is liberated through complement initiation) further promote this vascular penetrability by activating the kallikrein/kinin pathway and triggering the blood coagulation system [100]. This is depicted in Figure 5. Fatima et al. (2021) revealed that these mechanisms may be linked to gingival crevicular fluid (GCF) production and the headway of the inflammatory process, which ultimately contributes to severe alveolar bone damage at sites impacted by periodontitis [127].

Kgp (a product of the Kgp gene) is the most potent enzyme for degrading fibrinogen and fibrin among the three gingipains in human plasma [99]. It plays a role in the proclivity for bleeding seen in the unhealthy gingiva. Castro et al. (2017) reported that gingipains also target CD14 macrophages, inhibiting leukocyte activation via the lipopolysaccharide (LPS) receptor [128]. This action ultimately promotes the prolonged colonization of P. gingivalis [99].

Aggregatibacter actinomycetemcomitans

﻿Nørskov-Lauritsen et al. (2019) revealed that *Aggregatibacter actinomycetemcomitans* is recognized as a key pathogen in human periodontal disease, particularly in the confined form of insistent periodontitis [115]. Another study by ﻿Inoue et al. (2003) reported that the strain of *Aggregatibacter actinomycetemcomitans *310-A yielded a fimbrial antigen [129]. The amino acid sequence of the fimbrial protein was used to create oligopeptides [117]. ﻿Branching lysine polymer resin beads were used to link the peptide antigen. Melssen et al. (2022) reported that the peptide antigen was first diluted in phosphate-buffered saline (PBS), combined with incomplete Freund's adjuvant to create an emulsion. Finally, they injected into rabbits for immunization [119]. The rabbit antiserum showed reactivity against the fimbriae protein of *Aggregatibacter actinomycetemcomitans* 310a and other fimbriated strains containing a protein of about 54 kDa [120]. ﻿Fine et al. (2020) reported that the antiserum prevented fimbriated *Aggregatibacter actinomycetemcomitans* strains from attaching to saliva-coated hydroxyapatite beads, buccal epithelial cells, and a fibroblast cell line [130].

Hemagglutinins: Hemagglutinin B adhesion, which is non-fimbriated, appears to be a promising vaccine candidate. This protein plays a crucial role in bacterial adhesion and host cell invasion, as well as in the agglutination and lysis of red blood cells to acquire heme, an essential nutrient for bacterial growth [131]. Systemic and mucosal antibody responses were induced after intragastric administration of an avirulent *Salmonella typhimurium* strain carrying the hemagglutinin gene hagB in mice [132,133]. Furthermore, the antibody response could be enhanced, suggesting the activation of memory T cells or B cells [30].

Antigen: ﻿Carsetti and Quinti (2024) revealed that synthetic peptide adhesion between T-cell and B-cell epitopes is crucial for designing and evaluating synthetic peptide vaccines [134]. The role of adhesion epitopes in the immune response induced by synthetic peptide vaccines is highlighted by the involvement of IgG and secretory IgA, which may aid in preventing bacterial attachment to salivary glycoproteins or mucosal receptors [134]. Additionally, man-made protein molecules modeled on the nitrogenous matter structure have demonstrated the capability to inhibit *Porphyromonas gingivalis* from adhering to saliva-coated hydroxyapatite crystals [135].

GroEL heat shock protein: Heat shock proteins play a vital role in developing inflammatory responses, autoimmune disorders, and the advancement of atherosclerosis [136]. Research has demonstrated the presence of analogs of specific stress protein groups within the oral microbiota, including *Fusobacterium nucleatum*, *Prevotella *​​​​​​​*intermedia*, *Aggregatibacter actinomycetemcomitans*, and *Porphyromonas gingivalis*. Studies have confirmed the existence of analogous stress protein groups within the oral microbiota, including *Fusobacterium nucleatum*, *Prevotella ​​​​​​​intermedia*, *Aggregatibacter actinomycetemcomitans*, and *Porphyromonas gingivalis*. ﻿In their experimental research on rats, Lee et al. (2006) suggested that immunization with *Porphyromonas gingivalis *HSP60 reduced bone resorption caused by infections from various periodontopathic bacteria [137].

Passive Immunization

Passive immunity is conferred upon an individual through administering antibodies against a particular disease instead of eliciting an immune response from one's immune system [91]. ﻿Albrecht et al. (2022) that the transmission of passive immunity from mother to newborn occurs through the placenta [138]. People can gain passive immunity by receiving antibody-rich blood products, like immune globulin, particularly in cases where quick protection against a specific disease is needed [91]. Passive immunization is of limited duration due to the lack of host responsiveness to the immunization, resulting in transient protection corresponding to the persistence of the administered antibody [139]. In this process, antigens are introduced into a vector capable of generating antibodies, which induce passive immunization upon administration to a host. Two distinct methods can achieve this mechanism [140,141]: i. murine monoclonal antibodies, ii. plantibodies.

Passive immunization involves the administration of preformed antibodies to individuals deemed "at risk" [91] or during vulnerable intervals to disrupt microbial pathogenic mechanisms [91]. The concept of passive immunization for treating periodontal diseases has been explored due to the favorable results observed with both active and passive immunization against *Porphyromonas gingivalis* and *Streptococcus mutans*. Passive immunization is considered to carry a lower risk than active immunization [142].

A study by Okuda and colleagues in 1988 [143] showed that among small terrestrial mammals, *Porphyromonas gingivalis* colonization and synthesis of hemagglutinin in the periodontal area decreased when the animal-originated pre-formed antibodies that were applied ​​​​repeatedly.

When a T-cell clone specific to *Aggregatibacter actinomycetemcomitans* was isolated and transplanted into rats, it increased serum IgG and IgM antibody levels against *Aggregatibacter actinomycetemcomitans*, reducing bone resorption [61]. Therefore, the regulation of T-cells appears to contribute to the progression of periodontal disease, with T-cells, particularly CD4+ T cells, affecting alveolar bone destruction [144]. In their research, Cao et al. (2024) demonstrated that passive immunization with monoclonal antibodies targeting *Porphyromonas gingivalis* effectively inhibits the recolonization of this pathogen in humans [145].

Monoclonal antibodies have been used in passive immunization to combat periodontitis, demonstrating their ability to prevent the specific colonization of *Porphyromonas gingivalis* in humans. It is crucial to recognize that for a vaccine to be effective, it must stop the spread and/or intraoral growth of periodontal pathogenic microbes. An effective vaccine should ideally stimulate immunity at three levels [53]: i. resident mucosal secretory IgA, home-grown pump-off lymph nodes, and ii. precise T- and B-cell responses circulate in the body.

Promising outcomes from vaccination have included improved humoral immune responses, increased mucosal immunity, as evidenced by elevated IgA and IgG2 levels, and a decrease in *Porphyromonas gingivalis* and other subgingival microbiota species by preventing tissue infiltration and colonization of periodontal tissues [146]. Furthermore, the immunized group showed less bone loss than the non-immunized group, which was associated with lower prostaglandin E2 (PGE) levels in the gingival crevicular fluid [30].

Genetic Immunization

In the early 1990s, researchers initiated investigations into novel methodologies for developing vaccines characterized by distinct structural features compared to conventional ones. This method utilizes genetic manipulation techniques, such as genetic engineering and recombinant deoxyribonucleic acid (DNA) technology [7]. There are two types: i. Plasmid vaccines, and ii. Live, viral vector vaccines.

Plasmid vaccines: Plasmids can proliferate [147], unlike DNA. Due to this characteristic, plasmids can merge with the genetic material of a specific pathogen, facilitating the subsequent introduction into an organism to stimulate the production of antibodies [148,149]. The resultant product is subsequently administered to the recipient for immunization. Nevertheless, a disadvantage of plasmid-based vaccinations is the occasional risk of triggering oncogenic processes [150].

Live, viral vector vaccines: Proteins derived from pathogenic organisms exhibit a distinct structure that enables transcription within different pathogenic entities, including non-pathogenic DNA or ribonucleic acid (RNA) viruses and bacteria [151]. Once inside host cells, these carriers initiate protein synthesis, prompting a cellular or humoral immune response [152].

Routes of Vaccine Administration

Mucosal vaccination: Mucosal vaccines are generally more effective in protecting against periodontal disease than systemic vaccines, as they are better at simultaneously stimulating both IgG and salivary IgA in the mouth orifice [53]. All 11 preclinical studies examining this aspect found a two-fold increase in immunity within the oral cavity following mucosal vaccination, as opposed to systemic immunization. Moreover, some studies indicated that mucosal immunization offered protection against bone loss and gingival swelling associated with experimental periodontitis [53,153]. 

Oral immunization: Kwong et al. (2023) revealed oral vaccines are receiving increased attention because they are easier to administer, less invasive, generally safer, and more cost-effective than injectable vaccines [154]. In studies with rodents like mice, rats, and hamsters, oral vaccination targeting antigens from human periodontal infections led to robust antigen-specific antibody responses in both serum and saliva [155]. Oral gavage or intragastric intubation usually results in better levels of salivary IgA and a more considerable number of antibody-producing cells in mucosa-connected tissues compared to subcutaneous and intramuscular vaccination methods [6]. In addition, three studies investigated the clinical effects of these vaccinations on periodontal health, demonstrating defense against *Porphyromonas gingivalis*-induced alveolar bone loss or a reduction in gingival swelling in a mouse gingival abscess model [156-158].

Intranasal immunization: Intranasal vaccination provides benefits over oral immunization by avoiding the gastrointestinal breakdown of oral vaccines [159]. Studies on intranasal immunization for periodontitis in mice, rats, and canines revealed varying antigen-specific antibody reactions in both serum and saliva. Furthermore, in vitro analyses of the antibodies generated in eight studies showed protective effects, including reduced microbial assault of epithelial cells, lower biofilm development, and improved pathogen clearance [53].

Sublingual immunization: These studies showed that blood IgG levels were considerably lower after sublingual immunization than intranasal immunization, even though salivary IgA levels were similar [6,160,161]. Multiple studies indicated that sublingual immunization targeting this key pathogen considerably reduced mice's P. gingivalis-induced alveolar bone loss. However, intranasal intervention with the same vaccine offered more excellent protection [76,162].

Another article discusses the effects of the oral polymicrobial immunomodulator Dentavax (D), which is composed of killed bacterial and fungal cells (*Klebsiella pneumoniae*, *Streptococcus pyogenes*, *Staphylococcus aureus*, *Candida albicans*, and *Lactobacillus acidophilus*)*. *It is used for treating and preventing oral mucosal and periodontal inflammation. Key findings include: i. There are no significant changes in basic lymphocyte subsets; ii. Increased CD8 T effector cell activity and T-cell activation (CD69+) after immunization; iii. Strong lymphoproliferative responses, particularly with LPS+D and PHA+D co-stimulation; iv. Elevated TNF-alpha levels following stimulation with D; v. An increase in specific IgA, IgM, and IgG antibodies, especially IgA in saliva, indicates mucosal immunity. This study suggests that D stimulates lymphocyte function and enhances systemic and mucosal immunity, making it effective for immunoprophylaxis and therapy of oral inflammations [163].

Future Research Perspectives

Currently, around the globe, scientists are working to develop multiple pathogens and dysbiosis-controlling vaccines, induce protective immunity within the mouth cavity, enhance immunogenicity, and control the inflammatory process of the oral cavity triggered by periodontal diseases [17,146,164]. Additionally, pan-genes, enhancing host immunity, developing novel antimicrobial medications, and nanotechnology are also explored to mitigate the hostile outcome of periodontitis [165,166]. To maximize clinical outcomes, vaccines must be personalized based on individual genetics and microbiome profiles [167,168]. Multidisciplinary collaborations and standardized protocols must be fostered. Large-scale, long-term clinical trials must be conducted. Ethical and regulatory challenges in vaccine development must be addressed [169,170].

Limitations of the Narrative Review

Researchers could choose studies grounded on their individual understanding and foremost concern, perhaps initiating bias in the review [171]. Narrative reviews are frequently deficient in a robust protocol for examining, choosing, and evaluating studies, making it challenging to assess the superiority for substantiation [172]. Since narrative review follows a non-systematic method, the inferences of a narrative review probably may not be pertinent to a wider community [173]. Moreover, narrative review is often puzzling for other investigators to imitate the research findings [172]. However, narrative reviews possess the light to cover a wide range of topics, offering a wide-ranging impression of a research area. It also allows for exhaustive evaluation and clarification of research results and the power to recognize gaps in knowledge and produce novel research inquiries [174-176].

## Conclusions

Periodontal vaccines hold immense potential in the impediment and management of periodontal diseases. By targeting the root cause, microbial dysbiosis, vaccines offer a paradigm shift from conventional therapies focused on managing symptoms to addressing the underlying pathology. Although considerable advancements have been made in understanding potential antigens, delivery systems, and immune mechanisms, several challenges persist. These include antigen variability, the polymicrobial complexity of periodontal diseases, differences in host immune responses, and safety concerns like autoimmunity and cross-reactivity. The complexity of periodontal biofilms and their interaction with host immune systems necessitates a multidisciplinary approach, integrating advances in microbiology, immunology, and biotechnology. Future research should focus on novel antigen discovery, innovative delivery mechanisms, and personalized vaccination strategies to enhance efficacy. Furthermore, large-scale, long-term clinical trials and standardized protocols are crucial to corroborate the safety and effectiveness of these vaccines. Despite the challenges, the potential benefits of periodontal vaccines, like reduced disease burden, improved oral health, and broader systemic health implications, are compelling. Continued research and collaboration across scientific disciplines are crucial to overcoming the existing barriers and translating these innovative solutions into clinical practice. With sustained efforts, periodontal vaccines can revolutionize oral healthcare and improve quality of life globally.
